# Synthesis of ZIF-11 Membranes: The Influence of Preparation Technique and Support Type

**DOI:** 10.3390/membranes11070523

**Published:** 2021-07-12

**Authors:** Benjamin Reif, Jan Somboonvong, Martin Hartmann, Malte Kaspereit, Wilhelm Schwieger

**Affiliations:** 1 Institute of Separation Science and Technology, Friedrich-Alexander-Universität Erlangen-Nürnberg (FAU), Egerlandstraße 3, 91058 Erlangen, Germany; malte.kaspereit@fau.de; 2 Institute of Chemical Reaction Engineering, Friedrich-Alexander-Universität Erlangen-Nürnberg (FAU), Egerlandstraße 3, 91058 Erlangen, Germany; jan.somboonvong@fau.de (J.S.); wilhelm.schwieger@fau.de (W.S.); 3 Erlangen Center for Interface Research and Catalysis, Friedrich-Alexander-Universität Erlangen-Nürnberg (FAU), Egerlandstraße 3, 91058 Erlangen, Germany; martin.hartmann@ecrc.uni-erlangen.de

**Keywords:** ZIF-11 membrane, seeding and secondary growth, multiple in crystallization

## Abstract

Due to its structural features, ZIF-11 is one of the most interesting materials for gas separation applications. Herein, we report a systematic study on the synthesis of ZIF-11 as a supported membrane. For this, we adapted optimized conditions for the ZIF-11 powder synthesis, identified in our previous works, to form ZIF layers on symmetric and asymmetric stainless-steel and asymmetric α-Al_2_O_3_ supports. Different techniques were investigated for the challenging layer formation, namely, in situ crystallization (ISC), multiple in situ crystallization (MISC), and the seeding and secondary growth (SSG) method. It was possible to deposit ZIF-11 on different supports by ISC and MISC, although it was difficult to obtain complete layers. SSG, in turn, was more effective in forming dense and well-intergrown ZIF-11 layers. This agrees well with the generally accepted fact that seeding considerably facilitates layer formation. Systematic studies of both individual steps of SSG (seeding and secondary growth) led to a basic understanding of layer formation of ZIF-11 on the different supports. The best membranes prepared by rub seeding and secondary growth achieved Knudsen selectivity. Improved gas separation performance is expected if the formation of defects can be avoided.

## 1. Introduction

**Figure 1:**
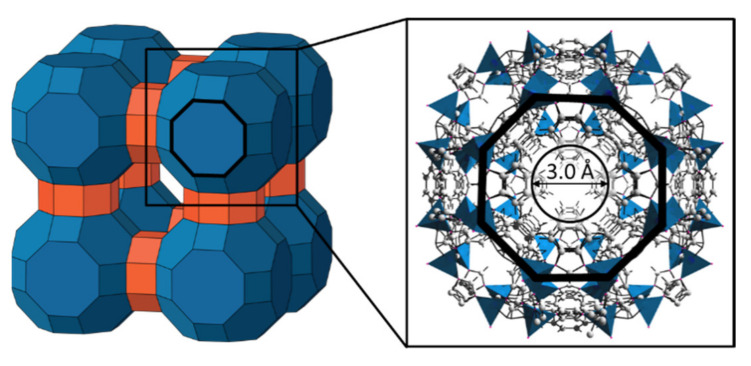
Schematic representation of the RHO topology of ZIF-11 (**left**) and detailed representation of the LTA unit and the resulting pore aperture (**right**).

In the last two decades, the group of zeolitic imidazolate frameworks (ZIFs) has become an integral part within the research area of porous membranes, especially when it comes to gas separation [1,2,3]. As a subgroup of metal–organic frameworks (MOFs), they offer unique properties such as high nanoporosity combined with small pore apertures, good thermal and chemical stability, and very regular pore structures [4,5,6,7,8]. The three-dimensional, crystalline network of ZIFs is built up by tetrahedrally coordinated transition metal nodes, e.g., Zn^2+^ or Co^2+^, which are linked by imidazolates. The combination of different metal nodes with a huge variety of imidazole derivatives enables tailoring the aperture or pore size toward any potential separation task. Compared to zeolites, which are the inorganic counterparts of ZIFs, synthesis of layers was brought to a new level by evolving a large toolbox of advanced synthesis methods for layers. This makes ZIFs even more interesting for membrane applications. Especially for the well-studied ZIF-8, methods like counter-diffusion [9,10], interfacial microfluidic processing [11], electrospray deposition [12], or synthesis based on the conversion of ZnO [13,14,15]—even in the absence of any solvent [16,17,18]—have been developed. In addition to ZIF-8, ZIF-11 is a very promising candidate for membrane applications such as hydrogen or natural gas purification. ZIF-11 possesses RHO topology and consists of Zn^2+^ cations connected by benzimidazolate (bIm^−^) linkers [4]. Compared to ZIF-8, ZIF-11 offers even smaller apertures (3.0 Å) and larger cages (14.6 Å) without compromising thermal and chemical stability (Figure 1).

There are different reasons that make ZIF-11 especially attractive for gas separation. Its structure combines large cavities with small six- and eight-ring pore openings, both being only slightly larger than the kinetic diameter of hydrogen (2.89 Å) [7]. First, the small pore openings suggest high molecular-sieving ability with respect to small molecules such as H_2_ and CO_2_, and the large cavities lead one to expect less mass transfer resistance as compared to structures offering smaller cavities. It should be noted that, as for all other ZIFs, the aperture diameter should be seen as a guide value rather than a fixed number. As highlighted in Figure 1, the pore opening diameter is defined by the phenyl groups of the benzimidazolate linkers. Thus, the generally accepted movement of those groups [19,20] leads to a diverging aperture. Another encouraging aspect is the uniformity of the RHO topology. This suggests isotropic mass transfer, i.e., the influence of crystal orientation within a substrate (as for mixed-matrix membranes) or on a substrate (as for pure ZIF layers) should not strongly affect mass transfer. Lastly, as already pointed out by Yaghi’s group [4] and further confirmed by He et al. [21] and Bennett et al. [22], ZIF-11 is amongst the most chemically and thermally stable ZIFs.

The separation potential of pure ZIF-11 layers and mixed-matrix membranes (MMMs) with dispersed ZIF-11 was also confirmed by molecular simulation studies, particularly for H_2_ and CH_4_ purification [23,24,25]. Thornton et al. predicted intrinsic gas permeabilities and separation properties for numerous ZIFs, and they found the highest H_2_ selectivity for ZIF-11 [25]. Yilmaz and Keskin studied the permeability of pure ZIFs and pure polymers in order to predict the performance of MMMs composed of different ZIFs and polymers [23]. They demonstrated that ZIF-11 possesses remarkable CO_2_/CH_4_ selectivity due to a large difference in CO_2_ and CH_4_ diffusion rates within the ZIF-11 pores.

According to these theoretical works, different groups succeeded in synthesizing ZIF-11 mixed-matrix membranes and confirmed their potential for gas separation. Thereby, ZIF-11 was incorporated in a number of different polymers such as polybenzimidazole [26,27], polysulfone [26,28], polyethersulfone [26], polyamide [29], Matrimid^®^ [30,31,32,33], polyimide (6FDA-DAM) [32,33,34], Torlon^®^ [32,33], and Pebax^®^ 2533 [35].

The group of Wang made progress in the synthesis of MMMs based on ZIF-11. They developed an efficient protocol for the room-temperature synthesis of micron-sized ZIF-11 using a toluene–ethanol mixture instead of *N*,*N*-diethylformamide (DEF) [4] at 100 °C [21]. By incorporating ZIF-11 in different polymers, they synthesized a promising ZIF-11/polybenzimidazole (PBI) MMM with a thickness of 15 µm, H_2_ permeability of 67.8 Barrer, and H_2_/CO_2_ selectivity of 5.0 (for w_ZIF-11_ = 16.1 wt.%) [26]. Higher ZIF-11 loadings led to higher permeability but lower selectivity. Compared to PBI, the compatibility of ZIF-11 with polysulfone (PS), as well as polyethersulfone, was poor, suggesting that membrane synthesis would not result in high-quality gas separation membranes.

In contrast to this finding, Guo et al. recently reported preparation of ZIF-11/PS MMMs (4–35 wt.% ZIF-11) with a thickness of 25 ± 1 µm that integrated nanosized ZIF-11 (average particle size 60 ± 16 nm) [28]. Nanosized ZIF-11 was synthesized according to the centrifugation method developed by the group of Coronas [30], which also uses a toluene–methanol mixture as the solvent as proposed by the group of Wang [21]. Interestingly, although using methanol as solvent which might be crucial for obtaining a solvent-free structure, as pointed out by Noguera-Díaz et al. [36], analysis of N_2_ sorption isotherms revealed a significantly lower pore volume of about 0.095 cm^3^·g^−1^ [28] as compared to ZIF-11 synthesized in DEF (V_Pore_: 0.33 to 0.43 cm^3^·g^−1^) [37].

As mentioned above, the group of Coronas optimized the ZIF-11 synthesis in a toluene–methanol mixture [21] toward formation of nanosized crystals (36 ± 6 nm) via the centrifugation route [30]. Different amounts of nanocrystals where then integrated into a polyimide Matrimid^®^ continuous phase (10 to 25 wt.%) to form MMMs with good adhesion between ZIF and polymer. The best membrane with 15 wt.% ZIF-11 showed promising results in the separation of H_2_ and CO_2_ at room temperature and 200 °C (P_H_2__ = 535 Barrer, α_H_2_/CO_2__ = 9.1). The group also extended the work of Li et al. [26] on PBI-based ZIF-11 MMMs [27] and succeeded in the addition of nanosized ZIF-11 into ultrathin polyamide membranes (supported on asymmetric polyimide P84^®^) for the nanofiltration of organic solvents [29].

Yumru et al. prepared ZIF-11/Matrimid^®^ MMMs with a thickness of 70 ± 10 µm and 10 to 40 wt.% micron-sized ZIF-11 crystals (0.2–2 µm) added to the polymer matrix [31]. In another work, the group studied the integration of ZIF-11 into polyimide 6FDA-DAM [34]. The same polymer in addition to Torlon^®^ and Matrimid^®^ was used by Forman et al., who applied multinuclear pulsed-field NMR spectroscopy to compare intracrystalline diffusivities of ethane and ethene for ZIF-11 embedded in MMMs and for ZIF-11 crystal beds [32,33].

Ehsani and Pakizeh reported the fabrication of ZIF-11 MMMs using the rubbery polymer Pebax^®^ 2533 as matrix and micron-sized ZIF-11 synthesized in a toluene–methanol mixture [35] according to He et al. [21]. Due to polymer rigidification and potential pore blockage, the incorporation of up to 30 wt.% ZIF-11 into the polymer matrix decreased the permeability of all measured gases apart from H_2_ [35]. At higher loadings (50% and 70%), voids formed inside the MMM and the permeability of all gases increased. For the membrane prepared with 50 wt.% ZIF-11, good adhesion between polymer and filler, as well as a significant CO_2_/CH_4_ selectivity of 12, was observed (α_H_2_/CO_2__ ≈ 0.3).

Despite the strong gas separation performance already achieved by ZIF-11 MMMs, as well as the promising predictions of molecular simulations, to the best of our knowledge, no pure ZIF-11 layer or membrane has been reported hitherto. Pure ZIF-11 membranes are of special interest because, first, the real potential of ZIF-11 membranes can only be studied on the pure material and, second, porous membranes offer significantly higher fluxes as compared to MMMs. We assume that the synthesis of pure ZIF-11 membranes is mainly hindered by the challenges in synthesis, more specifically, the strong influence of different synthesis parameters in the solvent *N*,*N*-diethylformamide, as discussed in our previous papers [37,38]. Therefore, as reviewed above, in most works, ZIF-11 synthesis was carried out in toluene–(m)ethanol mixtures, which is especially efficient for powder synthesis. According to our own investigations, we assume that, for layer formation, DEF might be the better choice since its lower vapor pressure in comparison to (m)ethanol and toluene enables slower drying, which helps to avoid the formation of defects. Furthermore, the formation of ZIF-11 in DEF is well studied and the regions of stable ZIF-11 formation were already determined [37]. Our kinetic studies revealed that ZIF-11 is a kinetic, i.e., metastable phase, which transforms to the nonporous phase ZIF-7-III with progressing reaction time, especially when the water content in the solvent DEF exceeds a certain level (0.3 wt.%). The most crystalline and porous ZIF-11 phase (pore volume V_P_ = 0.43 cm^3^·g^−1^) was formed already after 3 to 12 h of reaction at 60 °C with a Zn:DEF ratio of 1:1400 and a Zn:bIm ratio in the range between 1:8 and 1:15. The pore volume of ZIF-11 formed under those conditions was significantly higher than the pore volume of ZIF-11 synthesized in toluene/(m)ethanol mixtures (0.095 cm^3^·g^−1^ [28], 0.11–0.16 cm^3^·g^−1^ [30], 0.105 cm^3^·g^−1^ [34], 0.30 cm^3^·g^−1^ [36]). As shown by powder X-ray diffraction (PXRD), N_2_ sorption, and SEM, ZIF-11 underwent successive dissolution–recrystallization cycles together with heterogeneous nucleation at prolonged crystallization times, whereas neither crystallinity nor pore volume was enhanced compared to samples after 12 h of reaction. Our systematic studies and accompanying findings triggered us to have a closer look into ZIF-11 layer formation using the solvent DEF. Herein, we report the first pure ZIF-11 membranes synthesized by multiple in situ crystallization and seeding and secondary growth on porous α-Al_2_O_3_ and stainless-steel supports.

## 2. Materials and Methods

### 2.1. Materials

Zinc nitrate hexahydrate (Zn(NO_3_)_2_∙6H_2_O, 98%) and benzimidazole (bIm, C_7_H_6_N_2_, 99%) were purchased from Alfa Aesar (Germany) and were used without further purification. Toluene (Applichem Panreac, 99.5%), ethanol (Merck Emplura absolut), zinc acetate dihydrate (Zn(Ac)_2_∙2 H_2_O, Emsure Merck p.a.), and ammonia hydroxide (25 wt.%, Emsure Merck p.a.) were used without further purification. *N*,*N*-Diethylformamide (C_5_H_11_NO, >99%) was purchased from Merck (Germany) and was recycled after each synthesis batch. Therefore, the utilized DEF was collected in a Duran laboratory bottle and dried with molecular sieve 3A for at least 5 days. After drying, the DEF was transferred to a round-bottom flask, distilled under vacuum at 60 °C, and then kept in a Duran laboratory bottle until the next use. The water content of the purified DEF (w_H_2_O, DEF_) was checked by Karl Fischer titration and typically varied from 0.05 to 0.1 wt.%.

### 2.2. Synthesis Methods

#### 2.2.1. Synthesis of ZIF-11 Seed Crystals

Seeds were prepared in a mixture of toluene and ethanol according to the recipe of He et al. [21]. The synthesis was scaled up in order to obtain an adequate amount of seed crystals by using a 2 L Schott bottle as synthesis vessel. Then, 3.44 g (30 mmol) of bIm was dissolved in 207.30 g (4.5 mol) of ethanol, and 138.20 g (1.5 mol) of toluene and 2.04 g (30 mmol NH_3_) of ammonia hydroxide (25 wt.%) were added under continuous stirring (300 rpm). The synthesis was started after adding 3.29 g (15 mmol) of zinc acetate dihydrate. The molar composition of the synthesis solution was Zn:bIm:NH_3_:EtOH:toluene = 1:2:2:300:100. After 3 h of synthesis under stirring (300 rpm) at room temperature, seeds were removed from the synthesis solution by centrifugation. The powdery product was redispersed in ethanol and centrifuged two more times to remove any remaining reaction components. Drying was performed in a drying chamber at 70 °C for 12 h.

#### 2.2.2. Membrane Supports

In order to investigate the influence of support material and porositiy, four different membrane supports were used in this work. Table 1 gives an overview on the specifications. For each support, an abbreviation based on the manufacturer name was used throughout this work.

#### 2.2.3. Cleaning of the Membrane Supports

To remove any contamination, membrane supports were cleaned in boiling water for at least 1 h, followed by thoroughly cleaning with isopropyl alcohol and drying at 100 °C in a drying chamber for at least 12 h.

#### 2.2.4. In Situ Synthesis of ZIF-11 Membranes

In a typical synthesis, 15.0 g (148.29 mmol) of DEF was weighed into a 50 mL sample vial with a screw cap, which was used as the synthesis vessel. About 5.0 g of DEF was removed and used for dissovling 31.5 mg (0.106 mmol) of zinc nitrate hexahydrate in a 50 mL beaker. Then, 187.6 mg (1.59 mmol) of bIm was dissolved in the remaining DEF, and the vial containing the solution was put in an oil bath for at least 10 min (60 °C) while constantly stirring. Prior to synthesis, the magnetic stirring bar was removed and a cleaned support was put on the bottom of the sample vial containing the bIm–DEF solution. The synthesis was started by adding the zinc–DEF solution from the beaker. The molar composition Zn:bIm:DEF of the synthesis solution was 1:15:1400. After 6 h of synthesis, the supports were removed from the solution and carefully cleaned with a small amount of ethanol. After this, supports were dried for 12 h at room temperature followed by drying in vacuum for at least 1 h.

#### 2.2.5. Seeding and Secondary Growth of ZIF-11 Membranes

**Figure 2:**
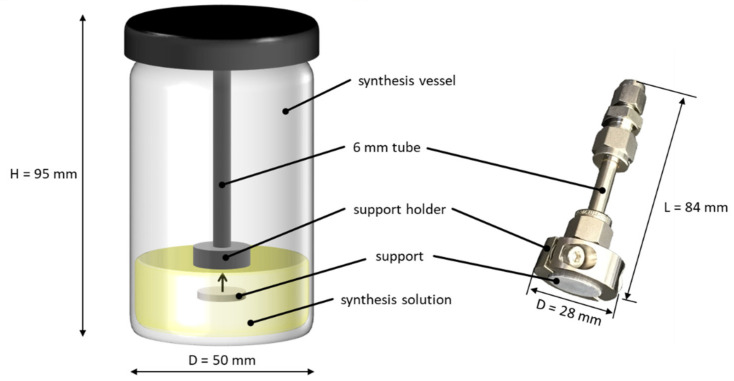
Schematic representation of the synthesis vessel with the support holder used for secondary growth (**left**) and image of the custom-made support holder (**right**).

Secondary growth was carried out in the same way as the in situ synthesis (Zn:bIm:DEF = 1:15:1400, 60 °C, 6 h) with seeded supports being added to the synthesis solution. For most syntheses, supports were not placed at the bottom of the vessel but placed hanging with the seeded side facing toward the bottom of the vessel. For this purpose, a custom-made stainless-steel support holder was used, which is shown in Figure 2.

By connecting the holder to a 6 mm stainless-steel tube via Swagelok fittings, the holder was mounted to the screw cap of the reaction vessel. The design of the holder was such that the surface of the support was not covered by the holder. Typically, the support was immersed about 10 to 20 mm below the surface of the synthesis solution.

#### 2.2.6. Seeding Procedures

**Figure 3:**
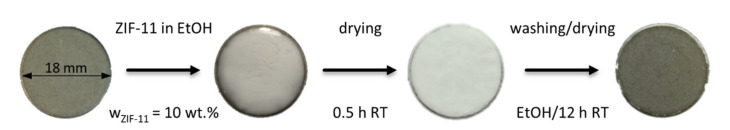
Images of a support (symmetric stainless steel, Mott Corporation) at different stages during drop seeding and typical parameters of drop seeding.

Different seeding procedures were applied in this work. The standard procedure was denoted drop seeding. For this, typically, 10 wt.% ZIF-11 seed crystals were dispersed in ethanol in an ultrasonic bath. For drop seeding, some of the dispersion was withdrawn with a Pasteur pipette and then applied dropwise onto the support such that the surface was covered completely. After drying at room temperature for 30 min, the coated support was transferred to a glass beaker filled with ethanol in order to specifically remove loosely attached crystals from the support surface. After that, supports were dried at room temperature for at least 12 h. Images of the support at different stages of preparation are shown in Figure 3.

For doctor blade seeding, a razor blade was drawn over the support surface to remove excessive seed crystals. For rub seeding [39], about a spatula tip of dry ZIF-11 crystals were put on a watch glass and then picked up by a finger covered in a nitrile glove. Then, an undefined amount of crystals was rubbed onto the support surface for about 10 s while applying slight pressure.

### 2.3. Characterization Methods

#### 2.3.1. X-ray Diffraction (XRD)

XRD patterns were recorded using a Philips X’PERT MPD diffractometer with CuKα radiation (40 mA and 40 kV) at a scan rate of 0.03°·(10 s)^−1^ and a step size of 0.02° between 2ϴ = 2° to 50°. The XRD patterns were compared to the reference patterns of ZIF-11 (CCDC number: 602545) and ZIF-7-III (CCDC number: 988184) in the Cambridge Structural Database [40].

The crystallinity was determined from the XRD results of the ZIF-11 membranes and is a measure of the amount of crystalline ZIF-11. For calculation, the sum of the integral intensity of a set of characteristic diffraction peaks of ZIF-11 was compared to that of a standard material (α-Al_2_O_3_ powder) measured in the same sample batch to minimize device-specific errors of the XRD. The analyzed reflecting angles were chosen as reported previously [37]. The integral intensity A_i_ of the sample was obtained by integration of the characteristic diffraction signals of the sample and the referring α-Al_2_O_3_ standard. The crystallinity was then calculated with Equation (1), where ‘*i*’ denotes the signal at the particular reflecting angle.

(1) $$Q_{\mathrm{Al}}^{\mathrm{ZIF}-11}=\frac{\sum_{i}{\;A}_{i}^{\mathrm{ZIF}-11}}{\sum_{i}{\;A}_{i}^{{\;\alpha -\mathrm{Al}}_{2}O_{3}}}.$$

#### 2.3.2. ZIF-11 Loading

ZIF-11 loading σ_ZIF-11_ (ZIF-11 mass per area) was calculated with Equation (2), where m_S_ is the mass of the support, m_S,ZIF-11_ is the mass of the support after ZIF-11 synthesis, and d_S_ is the diameter of the support (18 mm).

(2) $$\sigma_{\mathrm{ZIF}-11}=\frac{m_{S,\mathrm{ZIF}-11}\;{-\;m}_{S}}{\frac{\pi }{4}\cdot d_{S}^{2}}.$$

#### 2.3.3. Scanning Electron Microscopy (SEM)

SEM measurements of coated supports were performed using a GEMINI^®^ ULTRA™ 55 (Carl Zeiss) equipped with a Thermo Fischer EDX spectrometer. The SEM analyses were conducted using a secondary electron detector and an acceleration voltage of 1.0 kV.

#### 2.3.4. Permeance Measurements

Permeance measurements of single components were conducted at room temperature using a custom membrane cell and different volume flowmeters (e.g., Agilent ADM 2000, soap film bubble flowmeters). The membranes were sealed by using silicon flat gaskets from both sides of the membrane, and the effective remaining membrane area was 1.2 × 10^−4^ m^2^. All lines of the test rig were flushed with the pure single gas prior to measurements. For a measurement, the pure gas (H_2_, He, CO_2_, N_2_ or CH_4_) was loaded on the feed side with an absolute pressure of 2.0 bar and a typical flow rate of 150 mL·min^−1^. The permeate side was connected to the atmosphere, and the partial pressure of the measuring gas at the permeate side, as well as the partial transmembrane pressure, was 1.0 bar. After steady state was reached, the flow of the permeating gas was measured with the bubble flow meter, and the permeance was then calculated using Equation (3). $\pi_{i}$ is the permeance of component i in mol·m^−2^·s^−1^·Pa^−1^, ${\dot{V}}_{i}$ is the measured flow rate in m^3^·s^−1^, A_M_ is the effective membrane area in m^2^, Δp_i_ is the partial pressure difference across the membrane in Pa, and M_i_ and ρ_i_ are the molar mass and the density of the measured gas i in kg·mol^−1^ and kg·m^−3^, respectively.

(3) $$\pi_{i}=\frac{\rho \;\cdot {\dot{V}}_{i}}{A_{M}\;\cdot \;\Delta p_{i}\;\cdot {\;M}_{i}}.$$

## 3. Results and Discussion

In previous works [37,38] and, as discussed before, we identified the optimum conditions for a stable, reproducible synthesis of pure ZIF-11 powder. Herein, we demonstrate the adaptation of these synthesis conditions to prepare well-intergrown ZIF-11 layers. In the following, we describe the development of a preparation method starting from the simplest technique for layer deposition, which is in situ and multiple in situ crystallization (MISC). Subsequently, we move on to the more complex technique seeding and secondary growth (SSG).

### 3.1. In Situ and Multiple In Situ Crystallization

**Figure 4:**
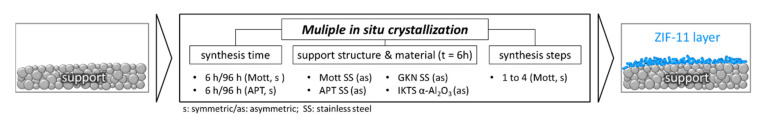
Overview on variations performed within multiple in situ crystallization of ZIF-11 layers.

Pure ZIF-11 powder is generally obtained from synthesis solutions with a molar ratio Zn:bIm:DEF = 1:15:1400 after 6 h at 60 °C [37]. Those conditions were applied to in situ crystallization of ZIF-11 layers unless indicated otherwise. Pre-dried DEF with a water content below 0.1 wt.% was used for all syntheses, since water accelerates the unwanted phase transition to the nonporous phase ZIF-7-III [38]. The successive dissolution–recrystallization of ZIF-11 indicated that repetitive, short syntheses should be used preferably rather than prolonged syntheses to avoid the dissolution of ZIF-11 formed on the porous supports. In order to confirm this hypothesis and to check on the influence of the support type, different supports were coated multiple times and with varying synthesis time. A schematic overview of the experiments is given in Figure 4.

A direct comparison of synthesis time (6 vs. 96 h) was performed for symmetric stainless-steel supports purchased from Mott Corporation (Mott) and Applied Porous Technologies (APT). The effect of the support structure, i.e., symmetric or asymmetric, was also investigated using asymmetric stainless-steel membranes purchased from GKN Sinter Metals (GKN), while the effect of the support material was studied on asymmetric α-Al_2_O_3_ supports from Fraunhofer IKTS (IKTS). Since it is known that multiple consecutive crystallization steps can have a positive effect on the membrane microstructure, multiple in situ crystallization, as proposed for zeolite membranes by Avhale et al. [41], was performed in greater detail for Mott supports. Table 2 summarizes the syntheses together with the synthesis parameters and the support specifications. The samples were named as follows: support name/number of synthesis step(s)/synthesis time.

#### 3.1.1. Influence of Synthesis Time on ZIF-11 Layer Formation

In a first set of experiments, symmetric APT supports were subjected to a ZIF-11 synthesis at 60 °C for either 6 or 96 h. No ZIF-11 was found on the supports whether by XRD or by weighing; thus, a second synthesis was performed.

**Figure 5:**
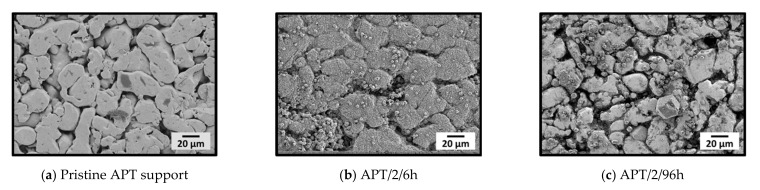
SEM images (**a**) of a pristine stainless-steel support (APT) and stainless-steel supports (APT) after two consecutive ZIF-11 syntheses for (**b**) 6 h and (**c**) 96 h in DEF at 60 °C.

Figure 5 shows the comparison of the SEM images of the twice-coated and the pristine APT supports. An almost complete covering of the support coated twice for 6 h was achieved, whereas only single, loosely attached crystals were present on the support coated twice for 96 h.

**Figure 6:**
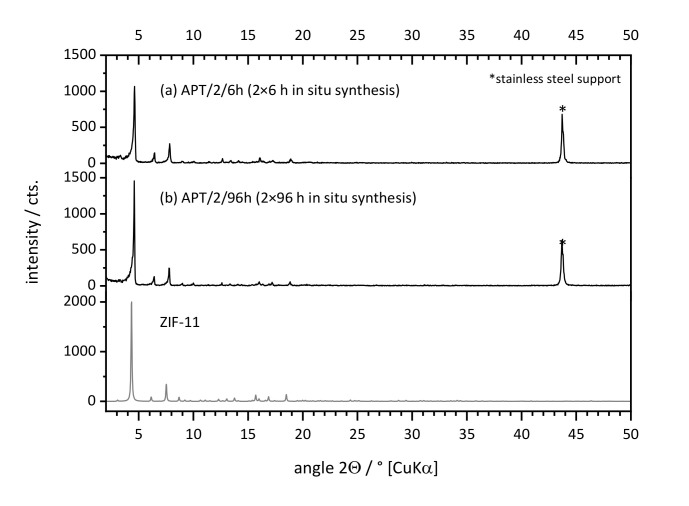
XRD patterns of two symmetric stainless-steel supports (APT) coated by two consecutive in situ syntheses in DEF at 60 °C for (**a**) 2 × 6 h and (b) 2 × 96 h, as well as PXRD reference pattern of ZIF-11.

The structure of the support was still easy to recognize for both membranes and, even for APT/2/6h, the pores were not completely covered by crystals. This was mainly due to the large pore openings of the symmetric supports used in this study, which are hard to cover completely. For APT/2/6h, defects and cracks were present within the layer, and a number of loosely attached crystals was found on top of it. Very likely, those crystals were formed by homogeneous nucleation and subsequent growth within the bulk of the synthesis solution and later settled on the support surface. The fact that ZIF-11 was detected only after two crystallization steps shows that the formation of nucleation sites on the supports is crucial. This in turn suggests that seeding may play an important role, as discussed later. As deduced already from the results of powder syntheses, short-term consecutive syntheses are much better suited for the formation of layers since successive dissolution and recrystallization is strongly pronounced for longer syntheses. XRD confirmed the presence of pure ZIF-11 in both cases (Figure 6).

The ZIF-11 loading σ_ZIF-11_, as well as the integral intensity of a set of characteristic diffraction peaks (hereafter referred to as crystallinity), was compared for both samples in order to estimate the quantity of deposited ZIF-11. As seen in Table 3, crystallinity and loading were nearly identical for both supports. This means that, for 6 h syntheses, a thin layer of well-intergrown crystals was formed, whereas 96 h syntheses led to the formation of the same amount of ZIF-11 in the form of larger, separate crystals and possibly a thin layer on the metal surface. It should be noted that the gas permeance (He, N_2_, CO_2_) through both layers was about equal to the permeance of a pristine support (3.5 × 10^−5^ mol·m^−2^·s^−1^·Pa^−1^) which means that no dense layer was formed in both cases.

#### 3.1.2. Influence of the Number of Synthesis Steps on Layer Formation

**Figure 7:**
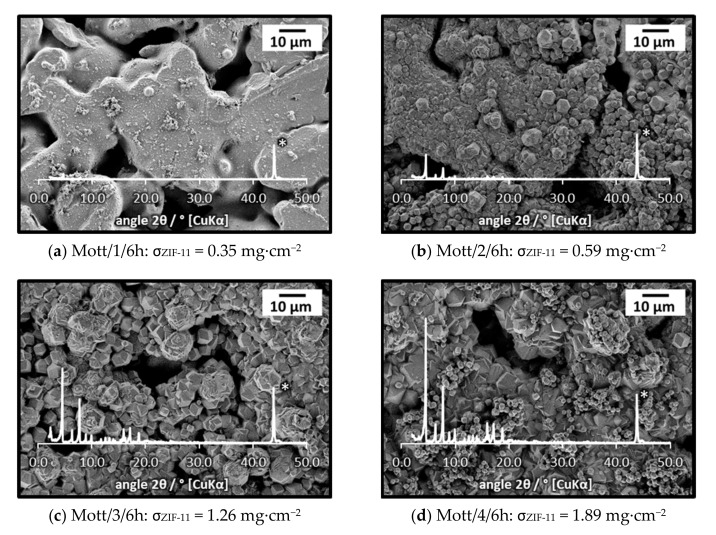
SEM images and XRD patterns of a stainless-steel (Mott) support at different stages of ZIF-11 multiple in situ crystallization in DEF at 60 °C (* support diffraction peaks).

Detailed investigations on the influence of synthesis number on the layer formation were carried out with stainless-steel supports purchased from Mott Corporation. For those supports, detectable amounts of crystals (Mott/1/96h: 0.47 mg·cm^−2^) were already present after one synthesis. As evident from by XRD and SEM (see Figures S1 and S2, Supplementary Materials), nonporous ZIF-7-III was formed after 96 h of synthesis. Thus, this synthesis was not investigated further. Instead, multiple in situ crystallization using consecutive 6 h syntheses was studied in detail. Figure 7 shows the SEM images together with the respective XRD patterns and ZIF-11 loading. After the first synthesis, the support surface was completely covered by a low amount (0.35 mg·cm^−2^) of small crystals and some larger agglomerates.

**Figure 8:**
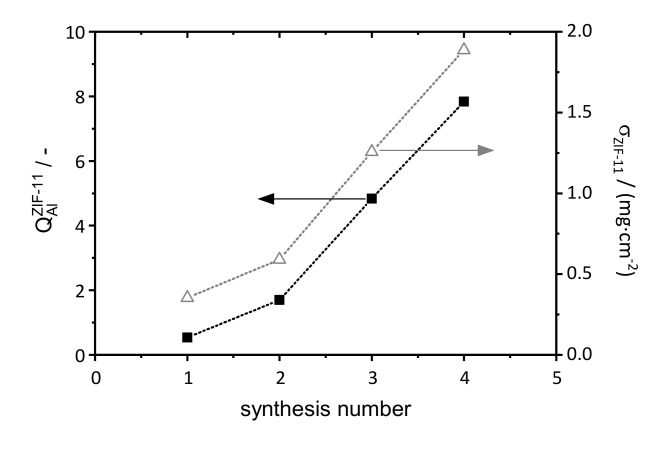
Crystallinity and ZIF-11 loading as a function of synthesis number during multiple in situ crystallization on a porous stainless-steel support (Mott Corporation).

The small crystals served as nucleation sites, and the ZIF-11 loading, as well as intensity of ZIF-11 diffraction peaks, increased significantly in the subsequent syntheses. Due to the large pore openings of the support, no dense layer was formed on the surface, but the coated regions were well intergrown and a deep-set ZIF-11 layer was visible inside the pores. Furthermore, sediments and cracks were visible in most of the layers. ZIF-11 loading and crystallinity are plotted as a function of the synthesis number in Figure 8.

Both curves followed the same trend and, thus, were appropriate values for quantitative analysis. The low initial gradient of both curves reflects the fact that the first synthesis was especially important to form seed crystals, which did not significantly increase the loading. After the seeds were formed, crystals grew and the deposited ZIF-11 loading increased linearly. The layer formation was accompanied by a slight reduction in permeance (Figure S3), even though the permeance stayed in the range of a pristine support (10^−5^ mol·m^−2^·s^−1^·Pa^−1^), thus confirming that no dense layer was formed even after four syntheses.

#### 3.1.3. Influence of Support Material and Porosity on Layer Formation

**Figure 9:**
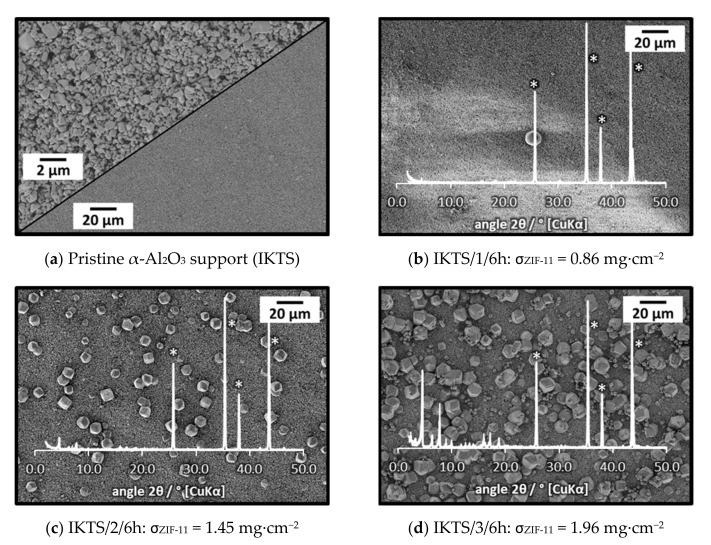
SEM images and XRD patterns of an α-Al_2_O_3_ support (Fraunhofer IKTS) at different stages of ZIF-11 multiple in situ crystallization in DEF at 60 °C (* support diffraction peaks).

As already mentioned, it was assumed that the main challenge in layer formation is covering the large pore openings of the support. Hence, asymmetric stainless-steel supports (GKN Sinter Metals), as well as α-Al_2_O_3_ (IKTS) supports, both with a very fine top layer (e.g., α-Al_2_O_3_, Figure 9), were treated in ZIF-11 synthesis solution.

Even after two in situ syntheses, only a very low amount of ZIF-11 was detected on GKN supports (Figure S4). On IKTS supports, the loading as function of synthesis number followed the same trend as for Mott supports (see σ_ZIF-11_ values in Figure 9), and the XRD patterns confirmed the formation of pure ZIF-11. In contrast, no layer but only single ZIF-11 crystals with a size of about 10 µm were formed even after three syntheses (Figure 9). Thus, the density of nucleation sites on α-Al_2_O_3_ IKTS supports was not high enough to enable layer formation. As frequently discussed in the literature, one way to circumvent this issue is the attachment of seed crystals prior to synthesis [42,43,44,45].

### 3.2. Seeding and Secondary Growth

**Figure 10:**
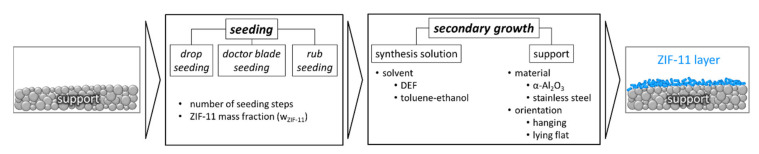
Overview on the variations performed for seeding and secondary growth of ZIF-11 layers.

Seeding and secondary growth was performed by applying ex situ seeding and secondary growth. The conditions of secondary growth complied with those of in situ synthesis (T = 60 °C, t = 6 h). We carried out a number of systematic variations for both seeding and secondary growth (Figure 10).

The samples were assigned according to the following systematic (see also Table 4): Support name/seeding procedure/support orientation/synthesis time.

Since seed deposition is crucial for forming a dense layer, different seeding methods were compared. Furthermore, the number of seeding steps, as well as the ZIF-11 mass fraction within the seeding dispersion, was varied. Secondary growth was also carried out in a mixture of toluene and ethanol. In order to study the influence of sedimentation, supports were placed either lying on the bottom of the synthesis vessel or hanging during secondary growth using a custom-made holder. Table 4 gives an overview on the specifications of the supports, as well as the sample names.

#### 3.2.1. Preparation of Seed Crystals

**Figure 11:**
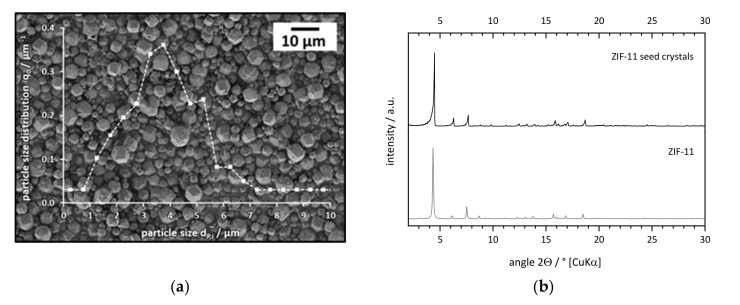
(**a**) Incremental particle size distribution (number) and (**b**) XRD pattern of ZIF−11 seed crystals prepared in a mixture of toluene and ethanol at room temperature.

ZIF-11 seed crystals were prepared in a mixture of toluene and ethanol according to the protocol of He et al. [21]. The room-temperature synthesis yielded highly crystalline, rhombic dodecahedral ZIF-11 crystals with an average particle size of 3.7 µm (Figure 11). As reported by other groups [28,30,34], the pore volume of 0.23 cm^3^·g^−1^, as measured by N_2_ adsorption at 77 K, was significantly lower compared to ZIF-11 prepared in DEF (V_P_ = 0.33–0.43 cm^3^·g^−1^ [37]), whereas the productivity of 3.9 mg_ZIF-11_·(g·h)^−1^ was much higher (DEF: 0.04–0.26 mg_ZIF-11_·(g·h)^−1^ [37]).

#### 3.2.2. Drop Seeding and Secondary Growth

**Figure 12:**
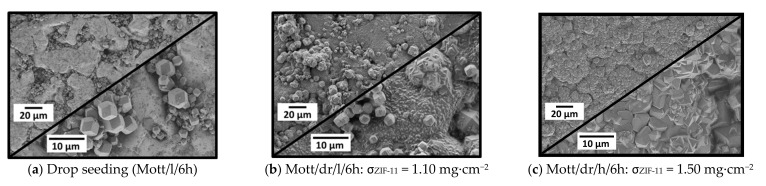
SEM images (500× and 2000×) of stainless-steel supports (**a**) after drop seeding and after secondary growth, (**b**) with support placed on the bottom of the synthesis vessel, and (**c**) with the support hanging with seeded side downward. Secondary growth was performed in DEF for 6 h at 60 °C.

A seed dispersion was prepared by dispersing 10 wt.% ZIF-11 in ethanol. Then, two symmetric stainless-steel Mott supports were drop-seeded and subjected to 6 h of secondary growth at 60 °C in DEF. To check if sedimentation could be avoided, support Mott/dr/l/6h was placed on the bottom of the reaction vessel as performed for in situ crystallization. In contrast, support Mott/dr/h/6h was placed hanging in the synthesis solution with its seeded side facing downward. The SEM images of both supports are shown in Figure 12.

The SEM image of the seeded support (Figure 12a) clearly demonstrates that the large pore openings of the support, which were shown to be difficult to cover by in situ crystallization, were almost filled completely with seed crystals, which is a major step toward coating dense layers. For both secondary growth syntheses, either with support placed on the bottom (Mott/dr/l/6h) or hanging in the synthesis solution (Mott/dr/h/6h), dense ZIF-11 layers were already formed after one synthesis, which shows the significant effect of seeding. The influence of support orientation is also clearly visible. While numerous single crystals and agglomerates were present on Mott/dr/l/6h (Figure 12b), which was placed lying on the bottom of the vessel, no sediments could be observed on Mott/dr/h/6h (Figure 12c). Consequently, supports were hung in the same way in all further experiments. Both layers seemed optically dense and consisted of well-intergrown crystals. The crystal size differed, i.e., larger crystals were formed on Mott/dr/h/6h. A SEM image of the cross-section of membrane Mott/dr/h/6h is shown in Figure S8.

**Figure 13:**
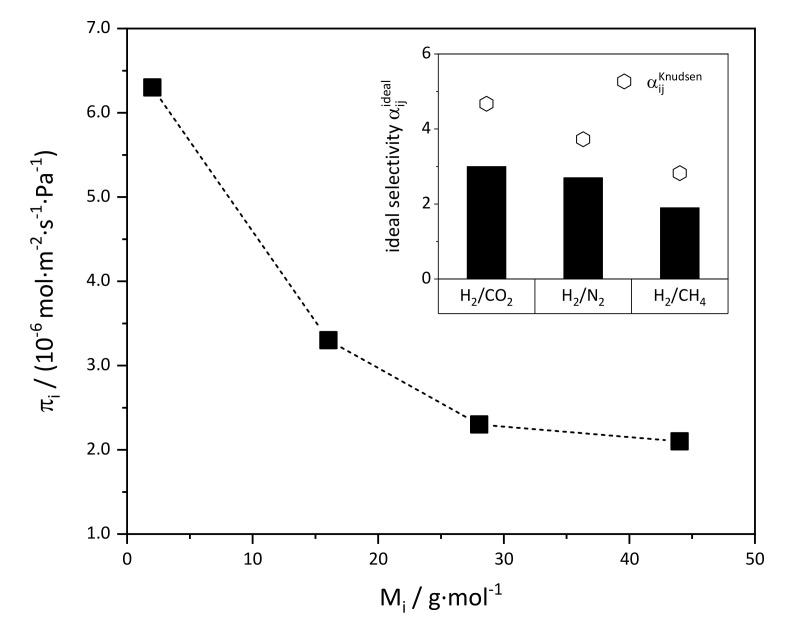
Single gas permeance (T = 25 °C, Δp = 1 bar) through a ZIF-11 membrane (Mott/dr/h/6h) as a function of the molecular mass of the measured gas M_i_. The membrane was prepared by drop seeding and 6 h of secondary growth in DEF at 60 °C.

Permeance measurements were performed with both membranes in order to assess the tightness of the membrane layers. Permeances of the measured gases were almost identical, and Figure 13 exemplarily shows the results for membrane Mott/dr/h/6h.

The permeance was a function of molar mass of the measured gas, which indicates that Knudsen flow significantly contributed to the overall flow. Compared to in situ membranes, the permeance was lower by about one order of magnitude. Thus, as already deduced from SEM images, the layers were denser and of higher quality compared to those prepared by in situ crystallization. However, selectivity was slightly lower than Knudsen selectivity, which means that larger nonselective pathways, i.e., defects such as cracks, were still present in the membrane layer.

Basically, as reported for other ZIF membranes [42,43], our results show that the performance of ZIF-11 membranes was significantly increased by seeding and secondary growth, although crack formation could not be avoided at this stage. It can be assumed that the membrane quality depends mainly on the condition of the seed layer, meaning that insufficient seed crystal coverage results in lower-quality membranes. For drop seeding, the influence of crystal coverage was studied in two different ways: First, by increasing the number of seeding steps and, second, by lowering the mass fraction of ZIF-11 in the seeding dispersion from 10 to 1.0 wt.%. While increasing the number of seeding steps had no effect on the crystal coverage and the resulting membranes (Figure S5), reducing the amount of ZIF-11 in the seeding dispersion had a negative effect on membrane quality. From this, it was concluded that the microstructure and, thus, the quality of the membranes can only be modified by changing the seeding method itself.

#### 3.2.3. Doctor Blade Seeding, Rub Seeding, and Secondary Growth

**Figure 14:**
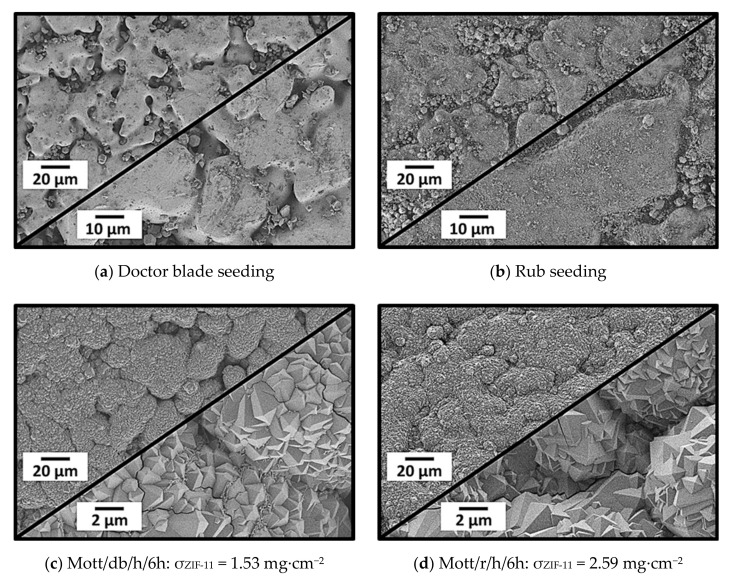
SEM images of stainless-steel supports (Mott) after (**a**) doctor blade and (**b**) rub seeding, as well as (**c**,**d**) the corresponding ZIF-11 layers after 6 h of secondary growth in DEF at 60 °C.

The influence of the seeding procedure was studied by applying doctor blade and rub seeding followed by secondary growth using symmetric stainless-steel supports. SEM images of the seeded supports, as well as after secondary growth, are shown in Figure 14.

Compared to drop seeding, fewer ZIF-11 crystals were present inside the pores of the support after doctor blade seeding (Figure 14a). However, at higher magnification, fine particle fragments were visible, which where most likely formed by shearing the seeds with the razor blade. For rub seeding, pores were completely filled with ZIF-11 seeds, and significantly more particle fragments covered almost the complete support surface. The small particles enhanced the formation of dense and well-intergrown layers. Due to the presence of several small seed fragments, the layers consisted of smaller crystals as compared to membranes prepared by drop seeding and secondary growth. The high ZIF-11 loading of 2.59 g·cm^−2^ of Mott/r/h/6h (Figure 14d) highlighted the positive effect of seeding and backed up the expectation that especially numerous small seeds are beneficial for layer formation.

**Figure 15:**
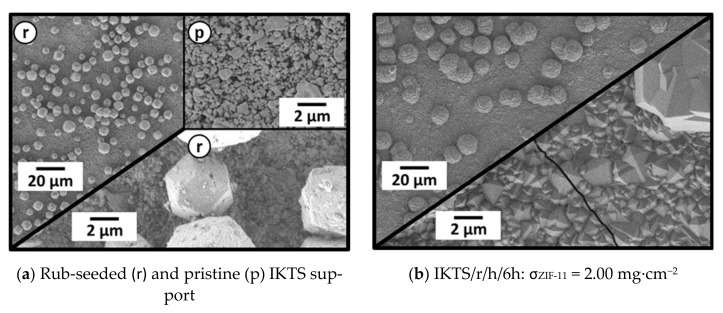
SEM images (500× and 5000×) of an IKTS support (α-Al_2_O_3_) (**a**) after rub seeding and (**b**) after 6 h of secondary growth in DEF at 60 °C.

Similar results were obtained for rub seeding and secondary growth using asymmetric IKTS (α-Al_2_O_3_) supports. Numerous seed crystals were present on the supports surface after rub seeding (Figure 15a). The direct comparison of the rub-seeded support to the pristine one at high magnification (see inset (p) Figure 15a) revealed that the entire surface was covered by a fine layer of seed fragments. After 6 h of secondary growth, a dense and well-intergrown layer was formed on the alumina support. This is in strong contrast to in situ synthesis, where only single crystals were formed on the support (Figure 9). The large seed crystals were integrated into the resulting layer, but in turn prevented the formation of a uniform layer. In order to form more even layers, either smaller seed crystals have to be used or the larger seed crystals have to be removed prior to secondary growth. At this point, the influence of the presence of large crystals on crack formation is not clear.

**Figure 16:**
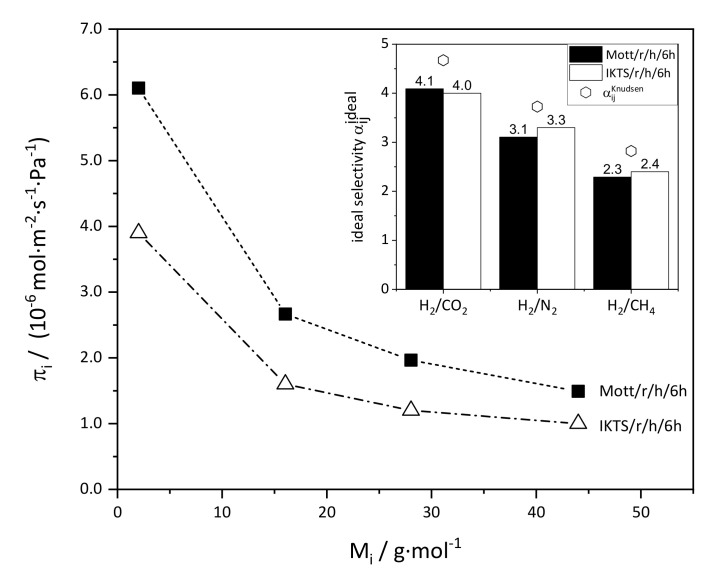
Single gas permeance (T = 25 °C, Δp = 1 bar) and ideal selectivity through ZIF-11 membranes as a function of the molecular mass of the measured gas M_i_. The membranes were prepared on symmetric stainless-steel (Mott/r/h/6h) and asymmetric α-Al_2_O_3_ supports (IKTS/r/h/6h) by rub seeding and 6 h of secondary growth in DEF at 60 °C.

However, regardless of the support, cracks were present in all layers after secondary growth. The effect of those cracks on permeance was studied exemplarily for membranes prepared on stainless steel and α-Al_2_O_3_ by rub seeding and secondary growth (Figure 16).

The permeance through both membranes was slightly lower as compared to the membranes discussed before, although some cracks were present. In turn, the selectivity was slightly higher. Furthermore, permeance was slightly lower for the membrane prepared on an IKTS support, which means that denser layers were formed. Mass transfer through both membranes occurred by Knudsen diffusion superimposed by viscous flow, which is not yet sufficient for application. However, the fact that the prepared defective ZIF-11 membranes already reached Knudsen selectivity suggests that defect-free ZIF-11 membranes offer great potential for gas separation. The results imply that α-Al_2_O_3_ supports are better suited to prepare uniform and possibly defect-free membranes when smaller seed crystals are used. The synthesis of nano-seeds may be performed via the centrifugation route described by Sánchez-Laínez et al. [30]. In order to compare the permeances of our membranes to those of MMMs including ZIF-11, we converted the reported permeabilities to permeances by using the reported membrane thicknesses. For example, for the MMMs reported by Li et al. [26], Yumru et al. [31], and Sanchez-Lainez et al. [30], the calculated permeances were in the range of 10^−10^ to 10^−9^ mol·m^−2^·s^−1^·Pa^−1^, while the H_2_/CO_2_ selectivities were not significantly higher or even similar. Of course, it has to be mentioned that non-defective ZIF-11 membranes are expected to have lower permeances, whereas higher selectivities can be assumed.

In order to verify those assumptions, defect-free ZIF-11 membranes have to be prepared, which in turn makes it relevant to study the origin of crack formation. This was not done in detail in this work. However, our results imply that crack formation can also be attributed to mechanical instability of the layers, especially when prepared on stainless steel. A short discussion on the initial results is given in the Supplementary Materials (see Chapter S5).

## 4. Conclusions

Dense and well-intergrown ZIF-11 layers were prepared on stainless-steel and α-Al_2_O_3_ supports for the first time, and we described in detail the individual steps from synthesizing pure ZIF-11 powder toward the preparation of ZIF-11 layers. The role of the preparation technique (MISC and SSG), as well as that of the membrane support materials and their porosity on the synthesis of ZIF-11 membranes, was studied systematically.

We first confirmed our hypothesis that, for the formation of layers by MISC, short, repetitive synthesis steps are suited considerably better than prolonged syntheses. This is due to (i) the successive dissolution and recrystallization during ZIF-11 synthesis and (ii) the significant influence of heterogeneous nucleation, which we showed for powders in earlier works. Furthermore, it was shown that both the material of the membrane support (α-Al_2_O_3_ and stainless steel) and its porosity play an important role in layer formation. Whereas MISC led to the formation of layers on symmetric stainless-steel supports, only single crystals were deposited on asymmetric α-Al_2_O_3_ and negligible amounts of ZIF-11 were formed on asymmetric stainless-steel supports. Detailed investigations on MISC using symmetric stainless-steel supports revealed that the synthesis number of MISC directly correlates with the amount of deposited ZIF-11. However, complete coverage of membrane supports was not achieved even after four repetitive syntheses.

In contrast, the seeding and secondary growth method yielded well-intergrown ZIF-11 membranes on both symmetric stainless-steel and α-Al_2_O_3_ supports. The best membranes were obtained when the supports were rub-seeded with ZIF-11 seed crystals and placed hanging in the synthesis solution, for which a custom device was developed. Although cracks could also be observed in these membranes, they already achieved Knudsen selectivity. It can be assumed that defect-free layers will exhibit significantly higher selectivity suitable for gas separation applications.

After successfully preparing pure ZIF-11 layers onto a variety of supports, the major future challenge for ZIF-11 membrane preparation is to focus on the reduction of defect formation. There exist a number of options to overcome the issue of crack formation. First, smaller seed crystals in combination with a more reproducible seeding method compared to rub seeding should be applied. Second, the influence of the drying conditions, as well as the mechanical stability of ZIF-11 layers, has to be studied. Lastly, the synthesis conditions during secondary growth (for example, the molar ratio of substrates) should also be varied in order to synthesize defect-free layers with high gas selectivity.

## Figures and Tables

**Table 1:** Overview on the membrane supports and their specifications used in this work.

**Cross Section**	**Symmetric**		**Asymmetric**	
	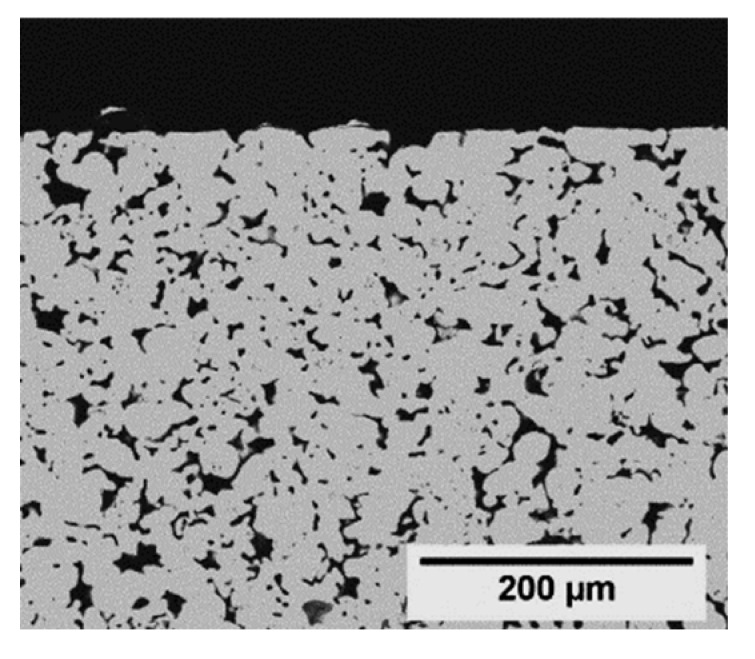 (Mott)		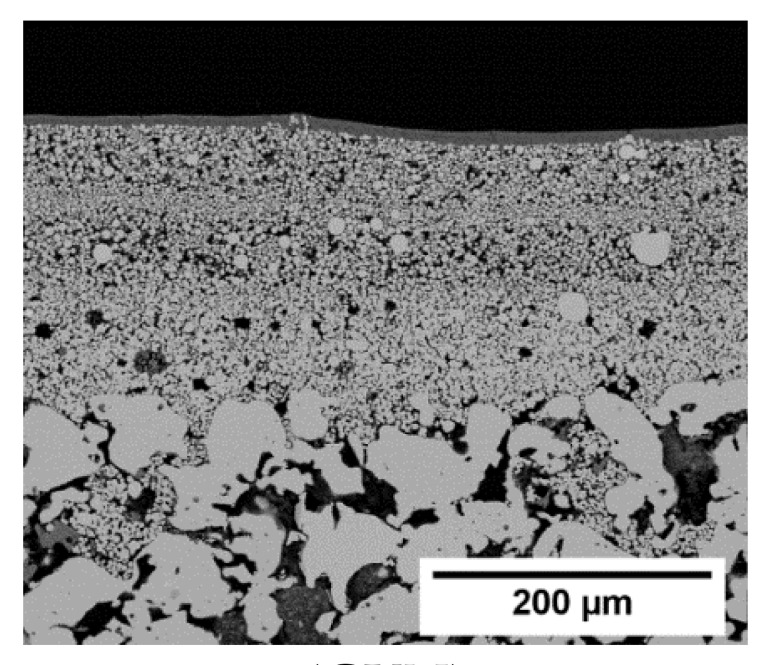 (GKN)	
Manufacturer	Mott Corporation	Applied Porous Technologies	GKN Sinter Metals	Fraunhofer IKTS
Support name in this work	Mott	APT	GKN	IKTS
Top view (image)	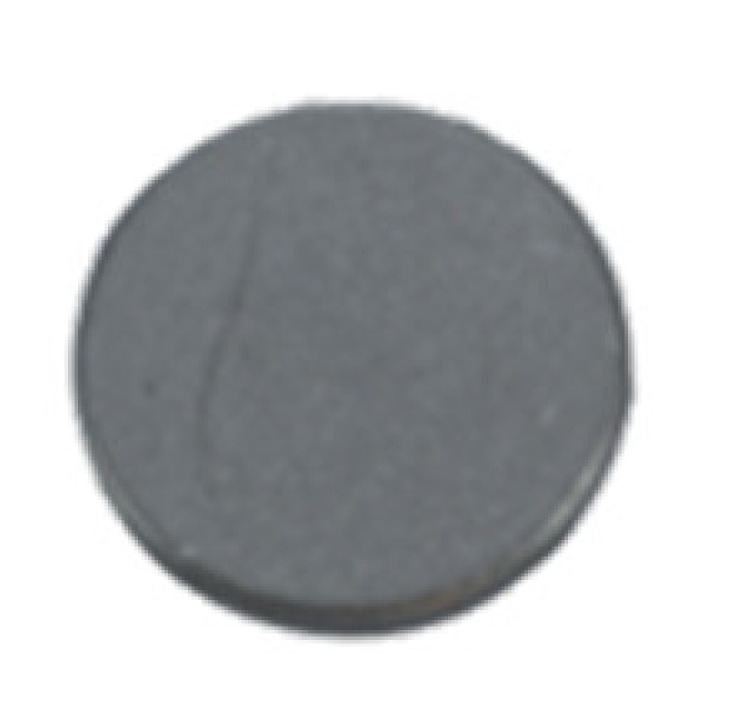	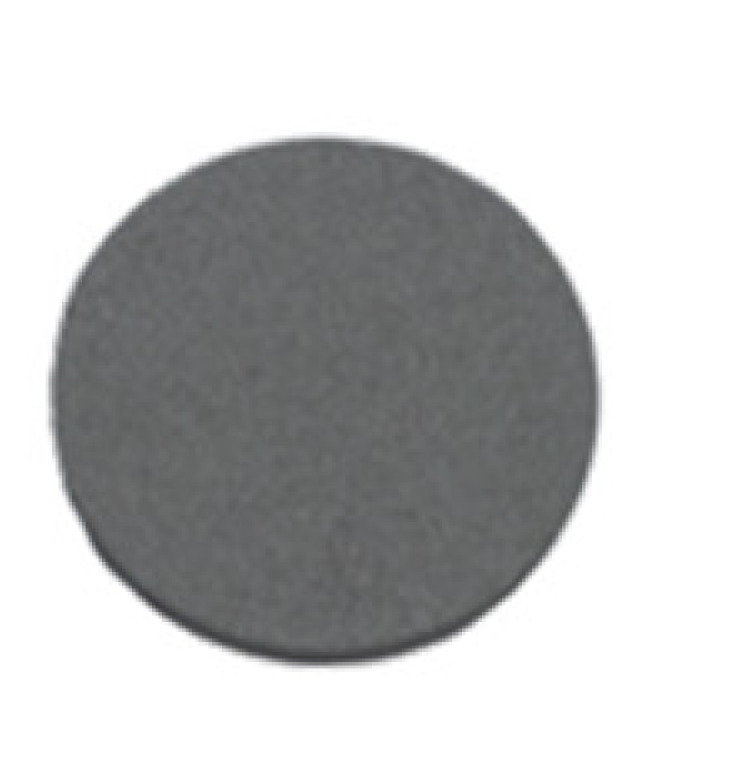	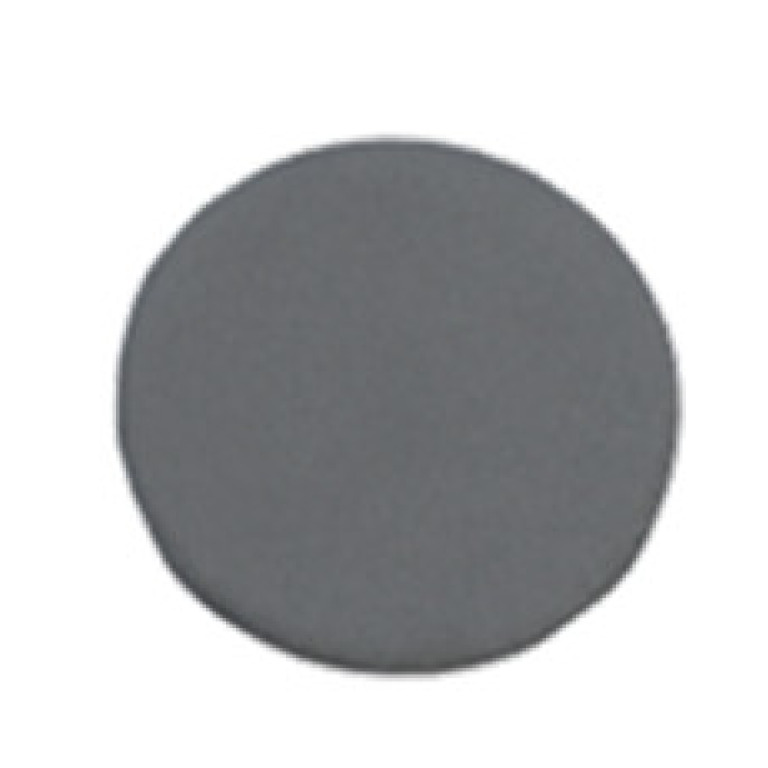	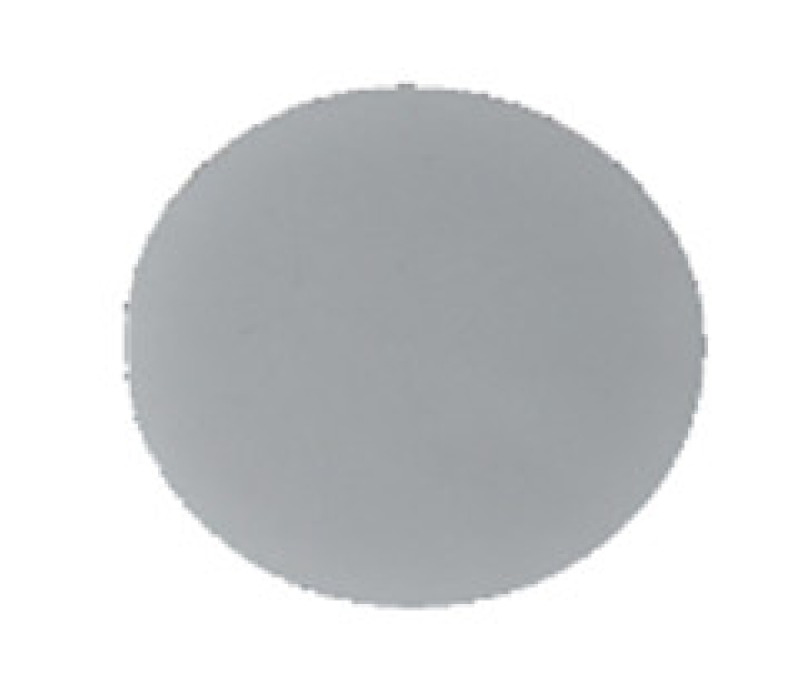
Material	Stainless steel ^a^	Stainless steel ^a^	Stainless steel ^a^	α-Al_2_O_3_
d_Pore_^b^ (µm)	0.2	0.1	0.1	0.2
Diameter (mm)	18.0	18.0	18.1	18.0
Thickness (mm)	2.0	2.0	2.0	2.0

^a^ AISI 316 L; ^b^ nominal pore size according to manufacturer.

**Table 2:** Overview on the different supports, their specifications and details on ZIF-11 deposition by in situ crystallization in DEF at 60 °C.

	Support Specifications			Synthesis	
Name	Material -	Type ^a^ -	d_P_^b^ (µm)	Number -	Time (h)
APT ^c^/2/6h	SS ^d^	s	0.1	2	6
APT ^c^/2/96h	SS ^d^	s	0.1	2	96
Mott ^e^/1–4/6h	SS ^d^	s	0.2	1 to 4	6
Mott ^e^/1/96h	SS ^d^	s	0.2	1	96
GKN ^f^/1–2/6h	SS ^d^	as	0.1	1 to 2	6
IKTS ^g^/1–3/6h	α-Al_2_O_3_	as	0.2	1 to 3	6

^a^ s: symmetric/as: asymmetric; ^b^ nominal pore size according to manufacturer; ^c^ Applied Porous Technologies; ^d^ stainless steel (AISI 316 L); ^e^ Mott Corporation; ^f^ GKN Sinter Metals; ^g^ Fraunhofer Institute for Ceramic Technologies and Systems.

**Table 3:** Comparison of crystallinity $Q_{\mathrm{Al}}^{\mathrm{ZIF}-11}$, the deposited ZIF-11 mass (m_ZIF 11_), and the ZIF-11 loading σ_ZIF-11_ on two APT stainless-steel supports coated two times for different reaction times.

Membrane	$Q_{\mathrm{Al}}^{\mathrm{ZIF}-11}$	m_ZIF-11_	σ_ZIF-11_
-	-	mg	mg·cm^−2^
APT/2/6h	4.0	4.50	1.77
APT/2/96h	4.2	4.70	1.85

**Table 4:** Overview on the ZIF-11 membranes prepared by seeding and secondary growth.

Name	Support Specifications		Seeding	Synthesis		
	Material	Type ^a^	-	Solvent	Support Position	Time/h
Mott ^b^/dr/l/6h	SS ^c^	s	drop (dr)	DEF	lying	6
Mott ^b^/dr/h/6h	SS ^c^	s	drop (dr)	DEF	hanging	6
Mott ^b^/db/h/6h	SS ^c^	s	doctor blade (db)	DEF	hanging	6
Mott ^b^/r/h/6h	SS ^c^	s	rub (r)	DEF	hanging	6
IKTS ^d^/r/h-6h	α-Al_2_O_3_	as	rub (r)	DEF	hanging	6
Mott ^b^/dr/hte/6h	SS ^c^	s	drop (dr)	toluene/EtOH	hanging	6

^a^ s: symmetric/as: asymmetric; ^b^ Mott Corporation; ^c^ stainless steel (AISI 316 L); ^d^ Fraunhofer Institute for Ceramic Technologies and Systems.

## Supplementary Materials

The following are available online at https://www.mdpi.com/article/10.3390/membranes11070523/s1: Figure S1. XRD pattern of a symmetric stainless-steel support (Mott Corporation) after one ZIF-11 in situ synthesis in DEF at 60 °C for 96 h and the PXRD reference patterns of ZIF 11 (CCDC number: 602545) and ZIF-7-III (CCDC number: 988184); Figure S2. SEM image of a symmetric stainless-steel support (Mott Corporation) after one ZIF-11 in situ synthesis in DEF at 60 °C for 96 h; Figure S3. Single gas permeance of He, N_2_, and CO_2_ (T = 25 °C, Δp = 1 bar) through a stainless-steel support after applying two, three, and four consecutive ZIF-11 in situ syntheses (t = 6 h) in DEF at 60 °C; Figure S4. XRD pattern of an asymmetric stainless-steel support (GKN Sinter Metals) after (a) one and (b) two ZIF-11 in situ synthesis steps in DEF at 60 °C (t = 6 h) and PXRD reference pattern of ZIF-11 (CCDC number: 602545); Figure S5. SEM images of a symmetric stainless-steel support (Mott Corporation) after different numbers of drop seeding (10 wt.% of ZIF-11 dispersed in Ethanol); Figure S6. SEM images (2000×) of a ZIF-11 membrane on a stainless-steel support (Mott) (a) before and (b) after mounting in the permeance measurement cell. The membrane was prepared by drop coating and 6 h of secondary growth at 60 °C in DEF; Figure S7. SEM image of ZIF-11 membrane Mott/dr/hte/6h prepared by drop coating and room temperature secondary growth (6 h) in a toluene–ethanol mixture; Figure S8. SEM image (1000×) of the cross-section of ZIF-11 membrane Mott/dr/h/6h. The ZIF-11 layer was prepared on a stainless-steel (Mott) support by drop seeding and secondary growth (6 h at 60 °C) with support hanging into the synthesis solution. The loading was 1.50 mg·cm^−2^.
