# Supplementary material for: Synthesis of ZIF-11 Membranes: The Influence of Preparation Technique and Support Type
Source: Membranes (Basel). 2021 Jul 12;11(7):523. doi: 10.3390/membranes11070523 (PMC8303830; doi:10.3390/membranes11070523)
Supplement: Supplementary file 1 [file membranes-11-00523-s001.zip › membranes-1279189-supplementary.pdf]

Supplementary Material

Synthesis of ZIF-11 membranes – The influence of preparation technique and support type

Benjamin Reif [1,2,*], Jan Somboonvong [1], Martin Hartmann [3], Malte Kaspereit [1], and Wilhelm Schwieger [2]

[1] Institute of Separation Science and Technology, Friedrich-Alexander-University Erlangen-Nürnberg (FAU), Egerlandstraße 3, 91058 Erlangen, Germany; jan.somboonvong@fau.de (J.S.); malte.kaspereit@fau.de (M.K.)

[2] Institute of Chemical Reaction Engineering, Friedrich-Alexander-University Erlangen-Nürnberg (FAU), Egerlandstraße 3, 91058 Erlangen, Germany; wilhelm.schwieger@fau.de

[3] Erlangen Center for Interface Research and Catalysis, Friedrich-Alexander-University Erlangen-Nürnberg (FAU), Egerlandstraße 3, 91058 Erlangen, Germany; martin.hartmann@ecrc.uni-erlangen.de

* Correspondence: benjamin.reif@fau.de; Tel.: +49-9131-85-27447

S1. In situ crystallization of ZIF-11 for 96 h on Mott supports

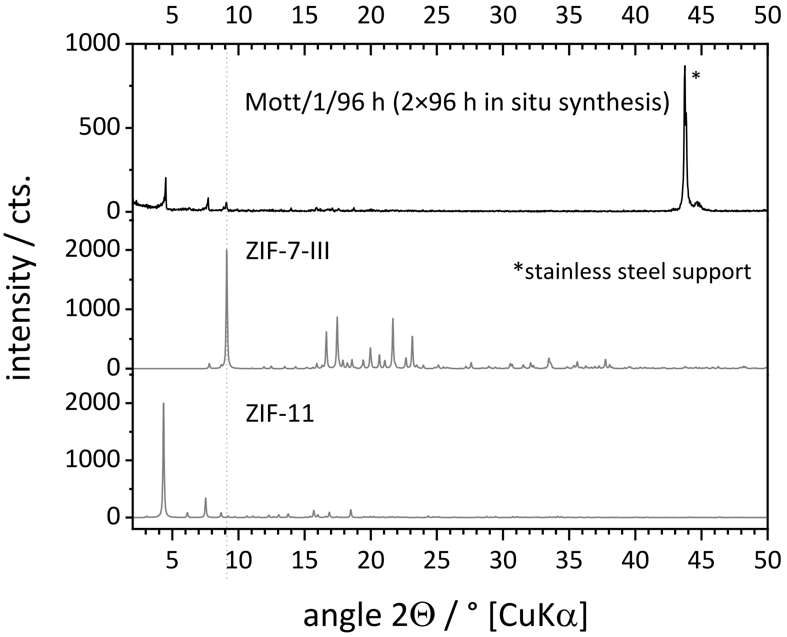

Figure S1. XRD pattern of a symmetric stainless steel support (Mott Corporation) after one ZIF-11 *in situ* synthesis in DEF at 60 °C for 96 h and the PXRD reference patterns of ZIF-11 (CCDC number: 602545) and ZIF-7-III (CCDC number: 988184).

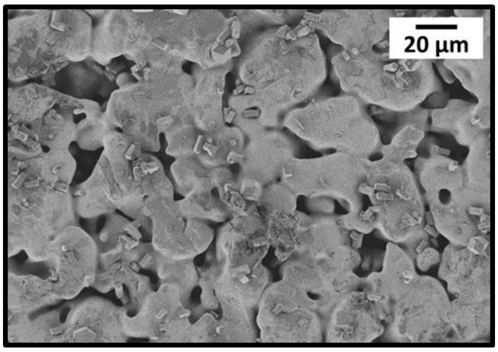

Figure S2. SEM image of symmetric stainless steel support Mott/1/96h (σ_{ZIF-11} = 0.47 mg·cm^{-2}) after one ZIF-11 *in situ* synthesis in DEF at 60 °C for 96 h.

S2. MISC (6 h, 60 °C) of ZIF-11 on Mott supports – Permeance

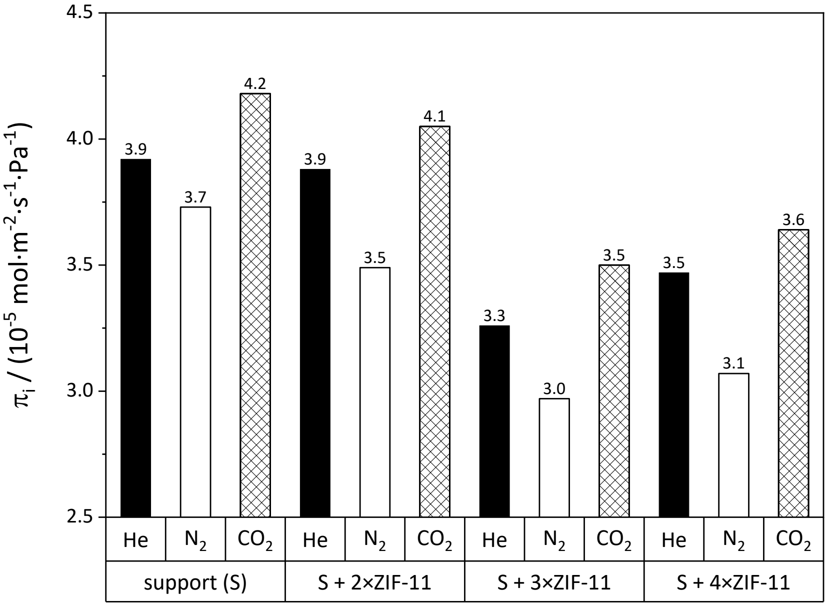

Figure S3. Single gas permeance of He, N_2 and CO_2 (T = 25 °C, Δp = 1 bar) through a stainless steel support after applying 2, 3 and 4 consecutive ZIF-11 *in situ* syntheses (t = 6 h) in DEF at 60 °C.

S3. In situ crystallization of ZIF-11 on GKN supports

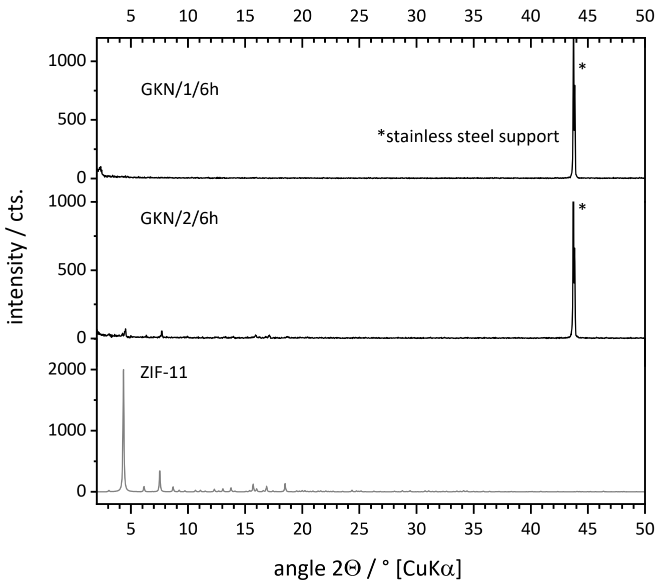

Figure S4. XRD pattern of an asymmetric stainless steel support (GKN Sinter Metals) after (a) one and (b) two ZIF-11 in situ synthesis step(s) in DEF at 60 °C (t = 6 h) and PXRD reference pattern of ZIF-11 (CCDC number: 602545).

S4. Results of repetitive drop seeding

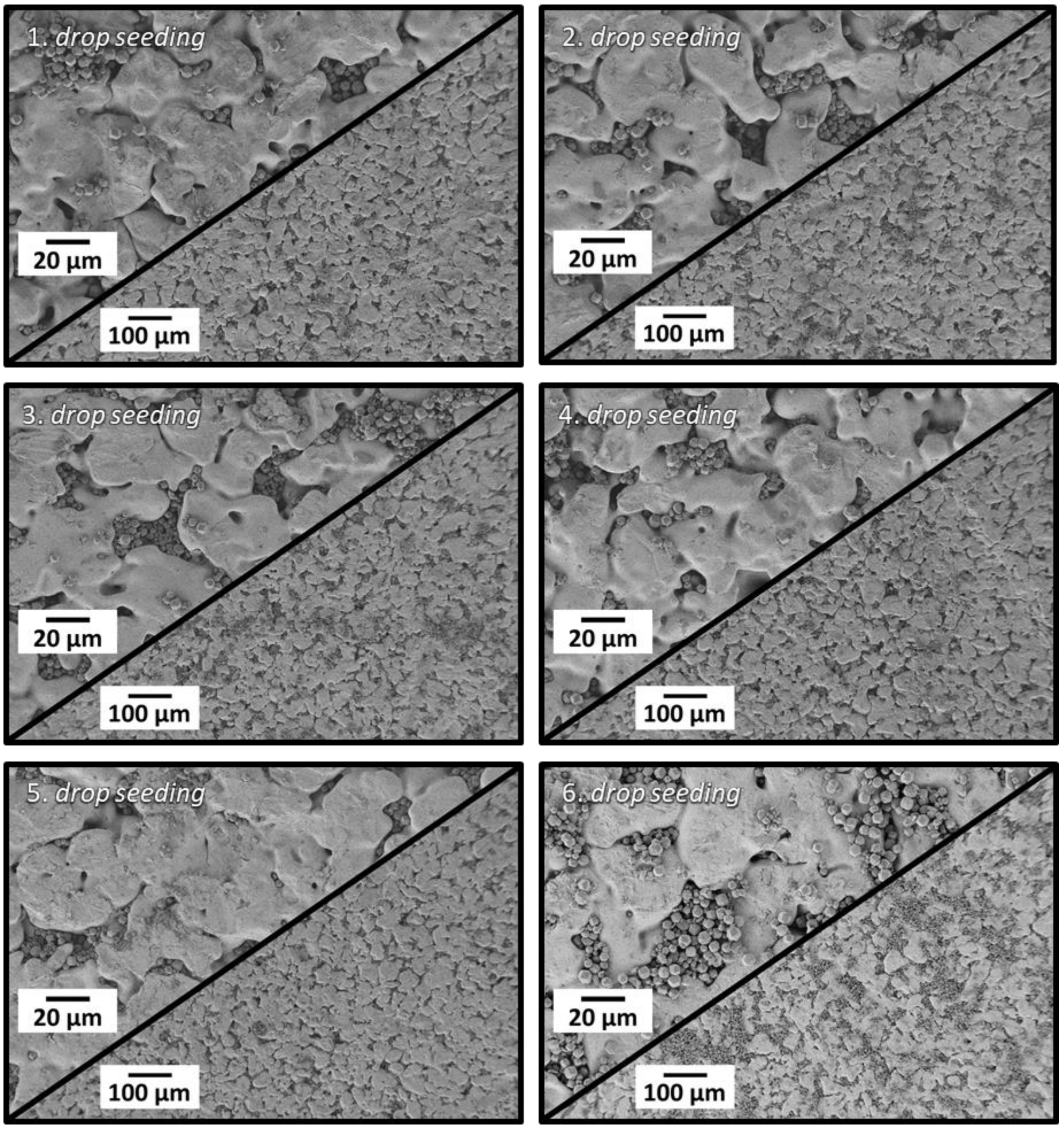

Figure S5. SEM images of a symmetric stainless steel support (Mott Corporation) after different numbers of drop seeding (10 wt.% of ZIF-11 dispersed in Ethanol).

S5. Short discussion on possible reasons for crack formation

During our investigations, we noticed that crack formation can also be attributed to mechanical instability of the layers. SEM images of a ZIF-11 membrane before and after permeance measurements show that the compressive force which is exerted by the sealing within the membrane test cell leads to defect formation (Figure S6).

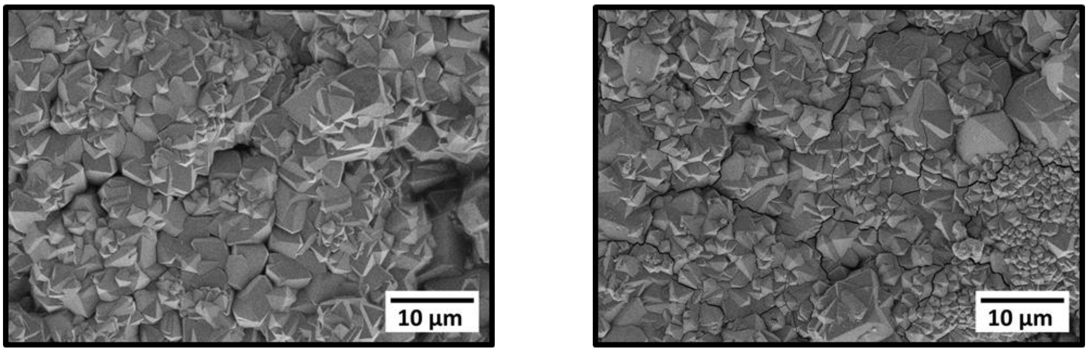

(**a**) ZIF-11 layer prior to mechanical stress (b) ZIF-11 layer after mechanical stress

Figure S6. SEM images (2000×) of a ZIF-11 membrane on a stainless steel support (Mott) (a) before and (b) after mounting in the permeance measurement cell. The membrane was prepared by drop coating and 6 h of secondary growth at 60 °C in DEF.

Furthermore, it is assumed that crack formation is facilitated by too fast evaporation of solvent as also discussed by Dong et al. on the example of ZIF-78 membranes [1]. This was confirmed by preparing ZIF-11 membranes in a mixture of toluene-ethanol which is lower boiling than DEF. Figure S7 depicts that the membrane exhibited numerous cracks and some parts of the support were not completely covered by the layer.

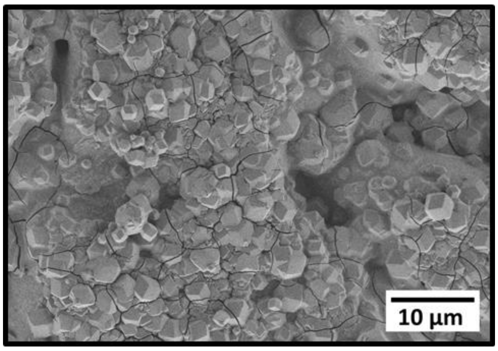

Figure S7. SEM image of ZIF-11 membrane Mott/dr/hte/6h prepared by drop coating and room temperature secondary growth (6h) in a toluene-ethanol mixture.

This also confirms our hypothesis that DEF synthesis is better suited for ZIF-11 membrane preparation. It should be noted that the drying of the membranes was not optimized within this work. Typically, membranes were not solvent exchanged but slightly rinsed with EtOH after synthesis and dried at room temperature. Possibly, optimizing the drying process will further enhance membrane quality.

1. Dong, X.; Huang, K.; Liu, S.; Ren, R.; Jin, W.; Lin, Y.S. Synthesis of zeolitic imidazolate framework-78 molecular-sieve membrane: defect formation and elimination. *Journal of Materials Chemistry* **2012**, *22*, 19222-19227, doi:10.1039/C2JM34102F.
